# Association of Handgrip Strength in Various Disabilities in Korean Adults over 50 Years Old: A Nationwide Cross-Sectional Study

**DOI:** 10.3390/ijerph19159745

**Published:** 2022-08-08

**Authors:** Yun-A Kim, Yoon Jeong Cho, Geon Ho Lee

**Affiliations:** Department of Family Medicine, Daegu Catholic University School of Medicine, Daegu 42472, Korea

**Keywords:** hand grip strength, disability, functional limitation

## Abstract

Several studies have shown an association between low hand grip strength (HGS) and functional limitations. This study aims to elucidate the association between HGS and functional limitations. We used the nationwide health examination data and included 13,517 Korean adults that were aged ≥ 50 years. We measured HGS using digital dynamometer and the maximum value of the dominant hand was divided into quartiles for the analysis. Functional limitations were assessed by using self-administered questionnaires. We categorized the 24 reported causes of functional limitations into musculoskeletal, cardiometabolic, neuropsychiatric, cancers, and others. In multiple regression analysis, the functional limitations tended to increase as HGS was lowered in both sexes. When analyzing according to the reasons of functional limitations, the ORs for functional limitations due to cardiometabolic problem tended to increase as the HGS decreased in men (*p* for trend = 0.039). Similar trends were observed in neuropsychiatric problem in women (*p* for trend = 0.002) and other problems in both men and women (*p* for trend = 0.014 in men, *p* for trend = 0.004 in women). No significant trends were observed for musculoskeletal problems and cancer in both men and women. The functional limitations were inversely associated with HGS, which were inconsistent according to different etiologies of functional limitations.

## 1. Introduction

Age-related loss of muscle strength and mass are important contributors to physical disabilities in older adults [1]. Reduced physical performance can lead to functional disability and an increased risk of falls, hospitalization, and mortality [2]. Among muscle strength measurements, hand grip strength (HGS) has been widely used because of its simplicity and inexpensiveness [3,4,5]. Grip strength is not only a surrogate of muscle function, but also a possible predictor of various clinical outcomes, such as disability, increased hospitalization, and mortality [6,7,8].

In a large prospective cohort study in older men, the lowest tertile of HGS was associated with an increased likelihood of disability after 25 years as 2.77 (95% confidence interval [CI] 1.70–4.54) compared to that which was observed in the highest tertile group [7]. In another 30-year follow-up cohort study in healthy middle-aged men, the relative risk of mortality was 1.25 (95% CI 1.08–1.45) in a normal weight group when comparing the lowest tertile group of HGS to the highest tertile group [9]. Recently, the relationship between HGS and quality of life has been studied. The likelihood of subjective poor general health increases 1.13-fold as HGS decreases per kilogram [10] and the relationship between HGS and quality of life differ according to sex and the quality of life [11]. While most studies regarding the clinical importance of HGS have been conducted in Western countries, Asian studies are relatively scarce [5,6,7,8].

The Korean elderly population aged > 65 years exceeded 14% of the total Korean population in 2017, which means that Korea had become an aged society; hence, this increasing trend is expected to continue to reach as high as 46.5% compared to 18.6% of the worldwide older population in 2067 [12]. Considering this aging phenomenon in Korea, examining whether HGS is associated with functional limitations in Korean middle aged and elderly population is worthwhile. Therefore, this study aimed to investigate the association between HGS and functional disability using Korean nationwide clinical data.

## 2. Materials and Methods

### 2.1. Data Collection and Study Participants

This study is a cross-sectional study which was based on data that were acquired from the Korea National Health and Nutrition Examination Survey (KNHANES) from 2014 to 2018. The KNHANES is an annually conducted nationwide cross-sectional health survey consisting of health interviews, health examinations, and nutrition surveys. A stratified, multistage, clustered probability sampling method was applied to this survey according to age, sex, and geographical area. All the participants provided informed consent for participation in the survey. The study was conducted in accordance with the Declaration of Helsinki and approved by the Institutional Review Board of Daegu Catholic University Medical Center (IRB No. CR-20-072).

Among the 39,199 participants who participated in the KNHANES from 2014 to 2018, we included adults that were aged ≥ 50 years who underwent HGS measurements and completed the self-reported questionnaire regarding functional disabilities. We excluded individuals who could not perform HGS measurement or did not complete the self-reported questionnaire regarding functional disabilities. Finally, 13,517 participants were included in the analysis.

### 2.2. HGS Measurements

Grip strength was measured using a digital hand dynamometer (digital grip strength dynamometer; T.K.K 5401, Japan), which reliability and validity had been already reported and the reported precision of the dynamometer was 0.1 kg [13,14]. Hand inspection and self-questionnaires were used to select individuals who were able to perform HGS measurements. In cases of missing the arm, hand, or finger, fractures of the finger, hand paralysis, and applied bandage or cast to the hand were excluded for HGS measurements. Individuals who had undergone any hand surgery within three months, a surgical history of arthritis or carpal tunnel syndrome, or experienced pain or stiffness of the hand within a week were also excluded from the HGS measurements.

Grip strength measurements were performed three times for each participant to reduce measurement errors. The participants were asked to stand upright with the elbow fully extended and squeeze the grip continuously at maximum force for at least 3 s. The dominant hand measurement was performed first, followed by the non-dominant hand. We used the maximal HGS among the recorded values for the statistical analyses.

### 2.3. Assessment of Functional Disabilities

Functional limitations were assessed by using a self-administered questionnaire. The participants who answered ‘yes’ to the question, “Do you have any limitations in daily living or social activities because of a current medical problem or physical or mental disorder?” were determined as having functional limitations, and participants who answered ‘no’ were categorized as not having functional limitations. Daily living and social activities include dressing, facial washing, bathing, self-feeding, ambulation, toileting, grooming, house-work, meal preparation, cleaning laundry, community mobility, use of communication devices, managing money, using the telephone, taking medications, meeting friends, and shopping for groceries, etc. 

The reasons for the functional limitations were also investigated. A total of 19 reasons in Wave 6 (2013–2015) and additional five reasons in Wave 7 (2016–2018) were presented as multiple choices. The participants were allowed to select all reasons for their functional limitations. We classified these reasons into five groups: musculoskeletal, cardiometabolic, neuropsychiatric, and cancer. Any injuries, including fracture or joint injury, arthritis, back or neck problems, obesity, and knee or leg pain were categorized as musculoskeletal problems. Heart disease, stroke, diabetes, hypertension, and renal failure were categorized as cardiometabolic problems. Depression, anxiety, dementia, and mental retardation were categorized as neuropsychiatric problems. Gastrointestinal disorders, dizziness, headache, respiratory problems or asthma, old age, dental or oral disease, vision problems, and hearing problems were categorized as others. 

### 2.4. Measurement of Covariates

We used the body mass index (BMI) data that were presented in the original KNHANES, which were calculated as body weight divided by the square of height in meters. Comorbidities were assessed using a self-administered questionnaire. The participants who answered that they had been diagnosed with stroke, heart disease, arthritis, depression, or cancer by a physician were considered to have comorbidities. Education level was also surveyed and categorized into four groups: elementary school or lower, junior high school, senior high school, and college or higher. Lifestyle habits were assessed by using a self-administered questionnaire. Physical activity was categorized as sufficient or insufficient according to the intensity and duration of weekly exercise. Sufficient physical activity was determined if the participants performed moderate-intensity physical activity for at least 150 min/week, high-intensity physical activity for at least 75 min/week, or an equivalent combination of moderate or high-intensity physical activity. If the participants consumed alcohol more than twice a week, with an average amount per drink of seven or more glasses for men and five or more glasses for women, they were considered high-risk alcohol consumers. The daily protein intake was also assessed using 24-h dietary recall questionnaire, and its amount was calculated and presented in the KNHANES data. Sufficient and insufficient protein intake was determined according to the dietary reference intake for Koreans [13]. 

### 2.5. Statistical Analyses

All analyses were performed using the SPSS software for Windows (version 21.0; SPSS Inc., Chicago, IL, USA). Sampling weights were used in all statistical analyses because of the complex sample design of KNHANES. Χ^2^ statistics for discrete variables and one-way analysis of variance for continuous variables were used to assess the differences between the characteristics according to sex and age. Continuous variables were presented as means with standard errors, and discrete variables as proportions with standard errors. HGS was categorized into quartiles for the analysis. Multiple regression analysis was performed to examine the association between the HGS and functional disability. Age, sex, BMI, education level, alcohol consumption, comorbidities, physical activity, and daily protein intake as a surrogate of nutritional status were adjusted to reduce a confounding bias. We also examined the associations between HGS and each of the five categories of functional disability according to the reported reasons, using multiple regression analysis.

## 3. Results

### 3.1. Characteristics of Study Participants

Table 1 shows the baseline characteristics of the study participants according to their sex. A total of 5989 men and 7528 women were included in this study. The majority of study participants were in their 50s. The average maximal HGS were 38.4 ± 0.1 kg in men and 23.1 ± 0.1 kg in women. Approximately 90% of the study participants were right-handed. More men than women reported having stroke and heart disease, although more women reported having arthritis, cancer, and depression than men. There were significant differences in education levels between the sexes. The proportion of former or current smokers and high-risk alcohol consumers was higher in men than in women. The proportion of participants of both sexes who performed aerobic physical activities was approximately 40%. Sufficient protein intake was observed more frequently in the men than in the women. Approximately 10% of the participants had functional disabilities, slightly higher in women than in men.

**Figure 1:**
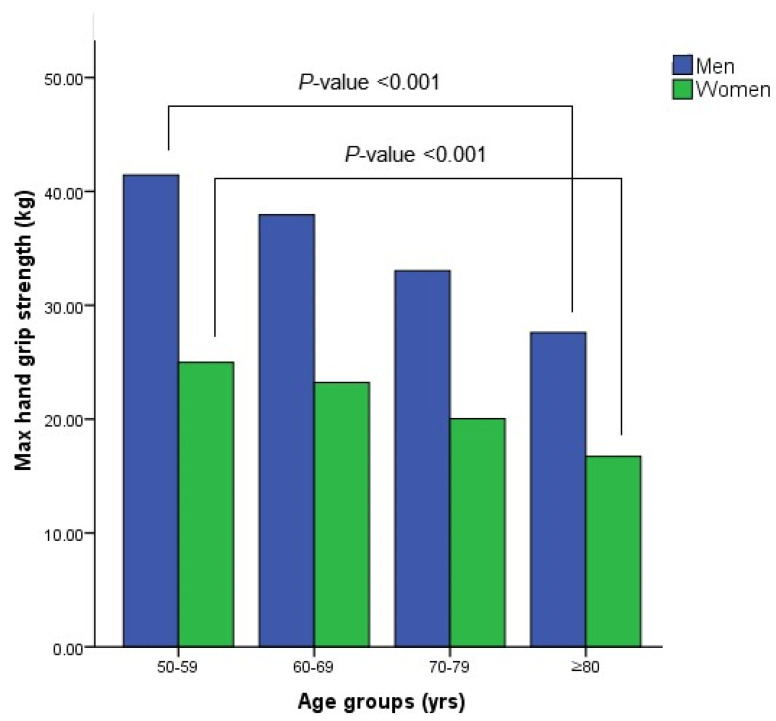
Distribution of hand grip strength according to age groups and sex.

Figure 1 shows the distribution of handgrip strength among the participants according to age group and sex. The HGS tended to decrease significantly as age increased, which was consistent in both men and women.

### 3.2. Functional Disability-Associated Factors according to Age Groups

Table 2 presents the various factors which can be associated with functional disabilities according to age groups. High-risk alcohol consumption tended to decrease as the age increased. Conversely, other factors, such as insufficient protein intake and insufficient physical activity tended to increase as the age increased. Regarding comorbidities, stroke, heart disease, arthritis, and depression tended to occur more as the age increased. However, a significant trend was not observed in cancer according to the age groups. The maximal HGS tended to decrease as age increased. 

### 3.3. HGS and Functional Disabilities according to Sex

Table 3 shows the odds ratios (ORs) and 95% CIs for functional disability according to quartiles of HGS and sex using multiple regression analysis. The cutoff values of HGS were 31.9, 37.2, and 42 kg in men, and 18.5, 22.3, and 25.8 kg in women, respectively. In men, the likelihood of functional disability was significantly higher in all the quartile groups than in Q1 (the highest quartile group). In Q4, the lowest quartile group, the likelihood of functional limitation was 2.82-fold greater than Q1 (OR,2.82; 95% CI:1.51-5.07). In women, the ORs for functional disability were 1.29 and 1.43% for Q3 and Q4, respectively, although statistical significance was not observed. However, the ORs for functional disability significantly increased as the HGS decreased in both men and women (*p* for trend = 0.002 in men, *p* for trend = 0.016 in women).

### 3.4. Associations of HGS with Different Etiologies of Functional Disability

Table 4 shows the associations between HGS and various reasons for functional disability according to sex, using multiple regression analysis. Regarding functional disability due to musculoskeletal problem, no significant associations were observed in both men and women according to HGS quartiles. In men, the likelihood of functional disability due to cardiometabolic problem in Q4 was 5.34-fold greater compared to Q1. In Q2 and Q3, the ORs for functional disability due to cardiometabolic problem were 1.53 and 2.45, although these were not statistically significant. The ORs for functional disability due to cardiometabolic problem significantly increased as the HGS decreased in men (*p* for trend = 0.039). In women, the likelihood of functional disability due to neuropsychiatric problem in Q3 and Q4 were 5.04 and 10.31-fold greater compared to Q1. Besides, the ORs for functional disability due to neuropsychiatric problems significantly increased as the HGS decreased in women (*p* for trend = 0.002). However, there were no significant associations between HGS and functional disability due to neuropsychiatric problem in men. Regarding functional disability due to cancers, no significant associations were observed in both men and women according to the HGS quartiles. Conversely, significant trends were observed between the HGS quartiles and functional disability due to other problems in both men and women (*p* for trend = 0.014 in men, *p* for trend = 0.004 in women). In men, the likelihood of functional disability due to other problems in Q4 was 3.50-fold greater compared to Q1. When the analysis was performed not divided by sex, functional limitations tended to increase as the HGS decreased in cardiometabolic, neuropsychiatric, and other problems (*p* for trends were 0.015, <0.000, and <0.000, respectively. Data not shown). In Q4, the likelihood for functional disability due to cardiometabolic, neuropsychiatric, and other problems were 7.92-, 8.81-, and 2.72-fold greater compared to Q1, respectively (data not shown). However, no significant associations with functional disability due to musculoskeletal problems or cancers were observed in any of the quartile groups (data not shown).

## 4. Discussion

In this study, we aimed to elucidate the association between HGS and functional disability using Korean nationwide clinical data. We observed that approximately 10% of the study population had a functional disability, which tended to increase as HGS decreased despite adjusted comprehensive confounding factors. Moreover, this inverse association differed according to the subjective reasons for functional disability and sex.

Functional impairment or disability originates from a progressive loss of function, although its concept has been variable [16,17]. Disability is a more comprehensive term that includes impairment, activity limitations, and restricted participation [18]. According to the Nagi model [19], disability is a social process that needs to be interpreted along with the surrounding environment, while functional limitation can occur when impairment can restrict physical performance. Recently, the “bio-psycho-social model” was developed by the World Health Organization International Classification of Functioning, Disability, and Health, which emphasizes environmental and personal factors in defining disability [18]. In this study, we assessed functional disabilities in daily living and social activities using a self-report questionnaire. The questions involved medical, physical, and mental problems as reasons for disability, which reflect the bio-psychosocial aspects of functional disability. Age, comorbidities, obesity, physical performance, muscle mass, and muscle strength have been suggested [16], and were mostly adjusted for the analyses in this study. 

Muscle strength is not only an important component for the diagnosis of sarcopenia [1], but has also been associated with negative consequences for clinical outcomes, such as postoperative complications [20,21,22], increased hospital stay [6,23], functional decline [7,24,25], and mortality [26,27,28]. As a muscle strength measurement, HGS has been strongly associated with overall muscle strength and is widely used as an indicator of muscle function [20] despite its limited role in the evaluation of lower extremity muscle function [29]. In our study, the likelihood of functional disability tended to increase as the HGS decreased in both sexes. The likelihood of functional disability was 2.82-fold greater in Q4, the lowest quartile HGS group, than in Q1, the highest quartile group in men. In women, the likelihood of functional disability was 1.29-fold and 1.43-fold greater in Q3 and Q4, respectively, than in Q1, although the difference was not statistically significant. These different results between men and women are aligned with previous findings that muscle weakness or muscle quality is more important for men to develop physical disability than for women [30]. In a meta-analysis of 12 HGS articles and functional status in older adults aged ≥ 60 years, a pooled ratio of functional disability was 1.78 (95% CI 1.28–2.48) when compared with the lowest versus the highest values of HGS [31]. When analyzing both men and women that were not classified by sex in our study, the likelihood of functional disability was 2.57-fold greater in Q4 than in Q1 (data not shown), which was similar to previous findings in epidemiological studies [7,25,32].

Notably, we observed that the association between HGS and functional disability differed according to subjective reasons for functional disability and sex. While a significant inverse trend was observed between HGS and functional disability due to musculoskeletal problems in men, this inverse trend was not observed in women. Conversely, there was a significant inverse trend between HGS and functional disability due to neuropsychiatric problems in women, and this relationship was not observed in men. Considering that most of the cardiovascular diseases such as myocardial infarction, angina, and stroke are more prevalent in men compared to women, and psychological disorders are more prevalent in women compared to men in Korea [33], we assumed that these might affect the different results according to sex. Among the five categories, the likelihood of functional disability due to cardiometabolic problems increased approximately 5-fold in Q4 compared to Q 1 in men. Moreover, the likelihood of functional disability due to neuropsychiatric problems increased approximately 10-fold in Q4 compared to Q1 in women. These associations that were observed in cardiometabolic and neuropsychiatric categories were relatively strong considering that the ORs of functional disability in Q4 were 2.82 (95% CI 1.57–5.07) in men and 1.43 (0.97–2.11) in women, respectively. The neuromuscular system plays a key role in maintaining physical independence, and reduced physical performance followed by physiological changes in the elderly muscular system can lead to functional disability [16]. In addition to physical performance, muscles are involved in various metabolic pathways and are used as a primary site for glucose uptake and homeostasis [2]. Muscles also interact with other organs via the synthesis and release of cytokines by myocytes [34]. Considering these various muscle functions and bio-psycho-social aspects of disability, we could assume that functional disabilities can develop through different pathways in the elderly population. 

However, we did not find a significant relationship between the HGS and musculoskeletal disability in both men and women. Since the muscular system is essential for mobility and daily movement that is needed for physical independence, we assumed that low HGS was associated with disability, especially in the musculoskeletal domain, similar to previous studies [35,36,37]. Since the distribution of HGS was significantly different according to each category of functional disability (*p* = 0.003, data not shown), this might have affected the unexpected results. While the average HGS were 27.9 ± 1.3 kg, 26.1 ± 1.2 kg, and 26.7 ± 0.5 kg in cardiometabolic, neuropsychiatric, and the “others” category, respectively, those were 25.7 ± 0.5 kg and 31.6 ± 1.2 kg in musculoskeletal and cancer category when analyzing the total study participants not divided by sex (data not shown). The relatively skewed HGS distribution of study participants and the small sample size for the cancer category could account for the non-significant associations between HGS and musculoskeletal or cancer-related functional disability that was observed in this study. 

This study had several limitations. First, we used a self-report questionnaire that was not validated as an assessment tool for functional disability. This might weaken the results of the study. However, the question assessing the functional limitations that were involved the evaluation of activities of daily living and instrumental activities of daily living, which have been widely used to assess functional status in previous clinical studies [31,38], and its validity and reliability already have been reported [39,40]. We also could not assess the severity of functional disability; thus, we could not evaluate whether functional disability was a preclinical or already manifested disability [41]. Lastly, because of the cross-sectional nature of the study, we could not assess the duration of functional disability and evaluate the causal relationship between HGS and functional disability. However, to the best of our knowledge, this is the first study to elucidate the association between HGS and functional disability in Korea. It is meaningful that we analyzed the causes of functional disability and found that HGS was associated differently according to the etiology of functional disability. Grip strength could affect different steps for functional disability differently. Moreover, we adjusted for various comprehensive confounding factors that could affect both HGS and functional disabilities, including nutritional status. In our study, there may be differences in the degree of influence depending on the cause of functional disability, but HGS could be considered as an indicator that can indirectly reflect the strength of muscles and functional status from various causes, especially in the elderly. Based on this research, we could suggest HGS measurement as one of the screening tools to find those who are at risk of functional limitations or have already had disability.

## 5. Conclusions

In conclusion, this study showed that HGS is negatively associated with functional disability in the middle-aged to elderly Korean population. Moreover, this association differed according to various etiologies of functional disability. These results suggest that HGS may be related to various pathways to the development of functional disability. Additional research is needed to elucidate the causal mechanisms between HGS and functional disability, to determine which step should be intervened and modified to prevent or reduce functional disability. 

## Figures and Tables

**Table 1:** Baseline characteristics of the study participants (N = 13,517).

	Men (N = 5989)	Women (N = 7528)	*p*-Value
Age group (yr)			<0.001
50 ≤ age < 60	49.4 (0.9)	44.4 (0.8)	
60 ≤ age < 70	26.3 (0.7)	28.8 (0.7)	
70 ≤ age < 80	19.1 (0.6)	20.7 (0.6)	
Age ≥ 80	5.3 (0.3)	6.2 (0.3)	
Height (cm)	167.6 ± 0.1	154.6 ± 0.1	<0.001
Weight (kg)	68.0 ± 0.2	57.7 ± 0.1	<0.001
Body mass index (kg/m^2^)	24.2 ± 0.1	24.1 ± 0.5	0.451
Maximal hand grip strength (kg)	38.4 ± 0.1	23.1 ± 0.1	<0.001
Dominant hand (%)			0.186
Right hand	87.8 (0.6)	89.1 (0.5)	
Left hand	5.4 (0.4)	4.6 (0.3)	
Both hands	6.9 (0.4)	6.3 (0.4)	
Comorbidities (%)			
Stroke	2.9 (0.2)	2.2 (0.2)	0.024
Heart disease	4.9 (0.3)	2.7 (0.2)	<0.001
Arthritis	7.6 (0.4)	26.4 (0.7)	<0.001
Cancer	1.7 (0.2)	2.8 (0.3)	0.001
Depression	1.7 (0.2)	4.5 (0.3)	<0.001
Education level (%)			<0.001
Elementary school or less	23.4 (0.8)	41.7 (0.8)	
Junior high school	16.2 (0.6)	16.8 (0.6)	
Senior high school	31.5 (0.8)	27.2 (0.7)	
College or more	28.9 (1.0)	14.3 (0.7)	
High-risk alcohol consumption (%)	16.9 (0.7)	0.3 (0.1)	<0.001
Sufficient physical activity (%)	40.1 (1.1)	37.2 (1.0)	0.036
Sufficient protein intake (%)	64.7 (0.8)	53.9 (0.8)	<0.001
Functional limitations (%)	9.0 (0.5)	11.6 (0.5)	<0.001

Abbreviations: NA, not available. Data are shown as mean ± standard error (SE) or proportion (SE).

**Table 2:** Different characteristics that were related with functional disability according to age groups.

	50 ≤ Age < 60	60 ≤ Age < 70	70 ≤ Age < 80	Age ≥ 80	*p* for Trend
High-risk alcohol consumption (%)	11.9 (0.6)	7.3 (0.5)	3.4 (0.4)	1.2 (0.5)	<0.001
Daily protein intake (g)	73.7 ± 1.1	65.0 ± 0.6	53.6 ± 0.6	45.5 ± 1.1	<0.001
Insufficient protein intake * (%)	34.1 (0.9)	39.8 (1.0)	51.5 (1.1)	65.1 (1.9)	<0.001
Insufficient physical activity † (%)	56.9 (1.2)	58.8 (1.3)	69.2 (1.4)	81.9 (1.8)	<0.001
Comorbidities (%)					
Stroke	0.9 (0.2)	2.4 (0.3)	5.4 (0.5)	5.7 (0.9)	<0.001
Heart disease	1.4 (0.2)	4.2 (0.4)	7.6 (0.5)	7.1 (0.9)	<0.001
Arthritis	8.6 (0.5)	21.5 (0.8)	29.4 (0.9)	26.9 (1.8)	<0.001
Depression	2.4 (0.3)	3.2 (0.3)	4.6 (0.4)	3.9 (0.8)	<0.001
Cancer	1.9 (0.2)	2.6 (0.3)	2.7 (0.4)	2.1 (0.6)	0.159
Maximal hand grip strength (kg)	33.5 ± 0.2	30.2 ± 0.2	26.3 ± 0.2	21.6 ± 0.3	<0.001

Data are shown as the mean ± standard error (SE) or proportion (SE). * Insufficient protein intake was determined according to the dietary reference intakes for Koreans [15]. The recommended protein intake is 60 g/day for men between 50 years and 65 years, 55 g/day for men over 65 years, 50 g/day for women between 50 years and 65 years, and 45 g/day for women over 65 years, respectively. † Sufficient physical activity was determined if the participants performed moderate-intensity physical activity for at least 150 min per week or high-intensity physical activity for at least 75 min per week or an equivalent combination of moderate or high-intensity physical activity.

**Table 3:** Multiple regression analysis * between hand grip strength ^†^ and functional disability according to sex.

	Men	Women
Q1	1.00	1.00
Q2	1.97 (1.23–3.17)	0.98 (0.67–1.43)
Q3	1.79 (1.04–3.08)	1.29 (0.88–1.89)
Q4	2.82 (1.57–5.07)	1.43 (0.97–2.11)
*p* for trend	0.002	0.016

Data are shown as odds ratios (95% confidence interval). * Adjusted by age, body mass index, education level, alcohol consumption, comorbidities, physical activity, and daily protein intake. Menopausal state was additionally adjusted for women. ^†^ Q1; the highest quartile group, Q2; the second quartile group, Q3; the third quartile group, and Q4; the lowest quartile group. Cutoff values were 42 kg, 37.2 kg, and 31.9 kg for men, 25.8 kg, 22.3 kg, and 18.5 kg for women, respectively.

**Table 4:** Multiple regression analysis * between hand grip strength ^†^ and each etiology of functional disability according to sex.

	Musculoskeletal	Cardiometabolic	Neuropsychiatric	Cancers	Others
Men					
Q1	1.00	1.00	1.00	1.00	1.00
Q2	1.46 (0.74–2.90)	1.53 (0.46–5.04)	1.06 (0.28–3.95)	7.00 (0.82–59.39)	2.36 (1.19–4.66)
Q3	0.99 (0.48–2.04)	2.45 (0.72–8.32)	1.33 (0.33–5.27)	3.61 (0.43–30.00)	1.86 (0.83–4.19)
Q4	1.46 (0.71–3.02)	5.34 (1.32–21.54)	2.26 (0.59–8.70)	1.45 (0.14–15.19)	3.50 (1.49–8.18)
*p* for trend	0.527	0.039	0.243	0.868	0.014
Women					
Q1	1.00	1.00	1.00	1.00	1.00
Q2	0.93 (0.60–1.45)	0.62 (0.17–2.21)	3.23 (0.59–17.77)	4.24 (0.65–27.70)	0.93 (0.47–1.82)
Q3	1.01 (0.65–1.56)	0.97 (0.42–2.22)	5.04 (1.03–24.67)	2.11 (0.25–17.53)	1.72 (0.94–3.12)
Q4	1.20 (0.77–1.85)	0.83 (0.27–2.57)	10.31 (2.04–51.97)	1.64 (0.07–37.87)	1.72 (0.95–3.10)
*p* for trend	0.261	0.924	0.002	0.927	0.004

Data are shown as odds ratios (95% confidence interval). * Adjusted by age, body mass index, education level, alcohol consumption, comorbidities, physical activity, and daily protein intake. Menopausal state was additionally adjusted for women. ^†^ Q1; the highest quartile group, Q2; the second quartile group, Q3; the third quartile group, and Q4; the lowest quartile group. Cutoff values were 42 kg, 37.2 kg, and 31.9 kg for men, and 25.8 kg, 22.3 kg, and 18.5 kg for women, respectively.

## Data Availability

Data that are not presented in the article are available upon reasonable request from the corresponding author.
